# Cardiopatia Chagásica: A Evolução da Doença e seus Exames Complementares

**DOI:** 10.36660/abc.20220418

**Published:** 2022-07-07

**Authors:** Claudio Leinig Pereira da Cunha

**Affiliations:** 1 Universidade Federal do Paraná – Clínica Médica Curitiba PR Brasil Universidade Federal do Paraná – Clínica Médica, Curitiba, PR – Brasil

**Keywords:** Doença de Chagas, Cardiomiopatia Chagásica/complicações, Evolução Clínica, Diagnóstico por Imagem/métodos, Epidemiologia, Insuficiência Cardíaca

Como em muitas áreas da Medicina, o cenário que abriga a Doença de Chagas (DC) tem mudado substancialmente. Há aproximadamente 50 anos, como Médico Residente, fazia plantões na Emergência do Hospital de Clínicas da Universidade Federal do Paraná e, rotineiramente atendia, em cada plantão, um ou mais casos de pacientes com franca Insuficiência Cardíaca Congestiva, anasarca, eletrocardiograma com distúrbios da condução e múltiplas arritmias, radiografia de tórax com cardiomegalia e diagnóstico laboratorial de DC. Não havia ecocardiograma. Hoje esta é uma situação incomum; quando ocorre, são chamados os estudantes para verem um quadro exuberante de Cardiopatia Chagásica (CC), que se apresenta a cada semestre.

Nos levantamentos epidemiológicos se observam constatações semelhantes. Em 1984 a prevalência da DC no Estado do Paraná, Brasil, era de 4% da população.^1^ Em 2020 as estimativas da prevalência de infecções pelo *Trypanosoma cruzi* foram de 1,02% a 2,4% no Brasil.^2^ No período 1975/83 entre 291 municípios paranaenses, 90 (30,9%) tinham insetos triatomíneos infestados pelo *T. cruzi*, enquanto que em 1990 estes foram encontrados em apenas 4 municípios (1,4%),^3^ com posterior erradicação da contaminação vetorial. As estratégias de controle vetorial têm propiciado um declínio substancial na prevalência mundial da doença, estimada em 18 milhões em 1990 e em 6 milhões em 2018.^4^

Apesar do evidente avanço na contenção de novos casos da DC, a população doente é ainda muito grande e exige cuidados especiais no seu diagnóstico e tratamento. No presente número dos Arquivos Brasileiros de Cardiologia, Tassi et al. estudam achados de exames complementares em relação às arritmias na CC.^5^

As técnicas laboratoriais para o diagnóstico da DC crônica não têm apresentado mudanças há anos. A antiga Reação de Machado-Guerreiro (fixação de complemento) não é mais empregada por sua baixa sensibilidade, baixa especificidade e complexidade na execução. São utilizados os testes de imunofluorescência indireta, hemoaglutinação e ELISA (imunoensaio enzimático). Tendo em vista a possibilidade de falso-positivos (leishmaniose, malária, sífilis, toxoplasmose, hanseníase, colagenoses, hepatites) é recomendado que o soro seja testado em pelo menos dois desses métodos para confirmação da positividade da sorologia. Na fase aguda da doença o teste preferido é a PCR (Reação em Cadeia da Polimerase).^6^

A avaliação cardiovascular dos pacientes com DC definida ou suspeita, é essencial para detectar os eventuais danos cardíacos. O eletrocardiograma é o teste mais importante na avaliação inicial, podendo indicar se já há uma cardiomiopatia instalada, presença de arritmias e contribuição para a estimativa do risco cardiovascular.^7^

A radiografia do tórax contribui na avaliação das câmaras cardíacas e da congestão pulmonar. O achado de cardiomegalia tem peso significativo na escala de risco de morte, proposta por Rassi, acrescentando 5 pontos para um máximo de 20.^8^

A ecocardiografia em geral é o teste chave, usado para identificar anormalidades estruturais e funcionais na DC. Integra a investigação de rotina, tanto na fase aguda como crônica, mesmo na Forma Indeterminada, e independente de sintomas. O estudo contribui para avaliação das funções ventriculares sistólica e diastólica, análise regional e global dos ventrículos esquerdo e direito, presença de aneurismas ventriculares, derrame pericárdico principalmente na fase aguda, pesquisa de trombos, regurgitações mitral e tricúspide, análise da hipertensão pulmonar.^9^

O Holter (Monitorização Ambulatorial do ECG) é outro exame fundamental para investigação diagnóstica, conduta terapêutica e avaliação prognóstica da DC. Permite estudar arritmias ventriculares complexas, fibrilação atrial, doença do nó sinusal e defeitos da condução atrioventricular e intraventricular.^10^

Pacientes selecionados com CC requerem avaliação adicional com outros exames: Teste Ergométrico, Coronariografia, Ressonância Magnética (avaliação ventricular em ecocardiogramas subótimos e pesquisa de fibrose), Testes de Medicina Nuclear (Ventriculografia radionuclear, SPECT, Imagem da inervação simpática miocárdica com MIBG-I123*, tomografia por emissão de pósitron com 18F-fluorodeoxiglicose*) e biópsia endomiocárdica* (* = aplicações em pesquisas).^4^

O estudo de Tassi et al.,^5^ exemplifica a evolução das pesquisas no entendimento das arritmias na CC.
