# Supplementary material for: Cardiopatia Chagásica: A Evolução da Doença e seus Exames Complementares
Source: Arq Bras Cardiol. 2022 Jul 7;119(1):12–3. [Article in Portuguese] doi: 10.36660/abc.20220418 (PMC9352126; doi:10.36660/abc.20220418)
Supplement: Translation

# Translation: Chagas Heart Disease: The Evolution of the Disease and its Complementary Exams

**Authors:** Claudio Leinig Pereira da Cunha

**Affiliations:** 1 Curitiba PR Brazil Universidade Federal do Paraná – Clínica Médica, Curitiba, PR – Brazil

**Keywords:** Chagas Disease, Chagas Cardiomyopathy/complications, Clinical Evolution, Diagnostic Imaging/methods, Epidemiology, Heart Failure

As in many areas of Medicine, the scenario that harbors Chagas Disease(CD) has changed substantially. For approximately 50 years, as a Resident Physician, I was on duty at the Emergency Department of the Hospital de Clínicas of the Universidade Federal do Paraná and routinely attended, on each shift, one or more cases of patients with clear Congestive Heart Failure, anasarca, electrocardiogram with conduction disorders and multiple arrhythmias, chest X-ray with cardiomegaly and laboratory diagnosis of CD. There was no echocardiogram. Today this is an unusual situation; when it occurs, students are invited to see an exuberant picture of Chagas’ heart disease (CHD), which appears every semester.

In epidemiological surveys, similar findings are observed. In 1984, the prevalence of CD in the State of Paraná, Brazil, was 4% of the population.^1^ In 2020, estimates of the prevalence of infections by Trypanosoma cruzi ranged from 1.02% to 2.4% in Brazil.^2^ In the period 1975/83, among 291 municipalities in Paraná, 90 (30.9%) had triatomine insects infected by T. cruzi, while in 1990, these were found in only 4 municipalities (1.4%),^3^ with subsequent eradication of vector contamination. Vector control strategies have led to a substantial decline in the global prevalence of the disease, estimated at 18 million in 1990 and 6 million in 2018.^4^

Despite the evident progress in the containment of new cases of CD, the sick population is still very large and requires care in their diagnosis and treatment. In the present issue of Arquivos Brasileiros de Cardiologia, Tassi et al. study the findings of complementary exams concerning arrhythmias in CHD.^5^

Laboratory techniques for the diagnosis of chronic CD have not changed for years. The old Machado-Guerreiro reaction (complement fixation) is no longer used due to its low sensitivity, low specificity and complexity of execution. Indirect immunofluorescence, hemagglutination and ELISA (enzyme immunoassay) tests are used. Because of the possibility of false positives (leishmaniasis, malaria, syphilis, toxoplasmosis, leprosy, collagen diseases, hepatitis), it is recommended that the serum be tested in at least two of these methods to confirm the positivity of the serology. In the acute phase of the disease, the preferred test is PCR (Polymerase Chain Reaction).^6^

Cardiovascular assessment of patients with definite or suspected CD is essential to detect eventual cardiac damage. The electrocardiogram is the most important test in the initial evaluation and can indicate whether there is already established cardiomyopathy, presence of arrhythmias and contribution to the estimation of cardiovascular risk.^7^

Chest radiography contributes to the assessment of cardiac chambers and pulmonary congestion. The finding of cardiomegaly has a significant weight in the risk of death scale proposed by Rassi, adding 5 points to a maximum of 20.^8^

Echocardiography, in general, is the key test used to identify structural and functional abnormalities in CD. It integrates routine investigation in the acute and chronic phases, regardless of symptoms, even in the Indeterminate Form. The study contributes to the assessment of systolic and diastolic ventricular functions, regional and global analysis of the left and right ventricles, presence of ventricular aneurysms, and pericardial effusion mainly in the acute phase, thrombus investigation, mitral and tricuspid regurgitation, analysis of pulmonary hypertension.^9^

Holter monitoring (ambulatory ECG monitoring) is another fundamental test for diagnostic investigation, therapeutic management and prognostic assessment of CD. It allows the study of complex ventricular arrhythmias, atrial fibrillation, sick sinus syndrome and atrioventricular and intraventricular conduction defects.^10^

Selected patients with CHD require additional evaluation with other tests: Exercise Tests, Coronary angiography, MRI (ventricular assessment on suboptimal echocardiograms and fibrosis research), Nuclear Medicine Tests (Radionuclear Ventriculography, SPECT, Myocardial Sympathetic Innervation Imaging with MIBG-I123*, positron emission tomography with 18F-fluorodeoxyglucose*) and endomyocardial biopsy* (* = research applications).^4^

The study by Tassi et al.^5^ exemplifies the evolution of research in understanding arrhythmias in CHD.
